# High-Fidelity NIR-LED Direct-View Display System for Authentic Night Vision Goggle Simulation Training

**DOI:** 10.3390/s25175368

**Published:** 2025-08-30

**Authors:** Yixiong Zeng, Bo Xu, Kun Qiu

**Affiliations:** School of Information and Communication Engineering, University of Electronic Science and Technology of China, Chengdu 611731, China

**Keywords:** NVGs, NVG training, NIR-LED display, night vision simulation, nonlinear response, inverse gamma correction

## Abstract

Current simulation training for pilots wearing night vision goggles (NVGs) (e.g., night landings and tactical reconnaissance) faces fidelity limitations from conventional displays. This study proposed a novel dynamic NIR-LED direct-view display system for authentic nighttime scene simulation. Through comparative characterization of NVG response across LED wavelengths under ultra-low-current conditions, 940 nm was identified as the optimal wavelength. Quantification of inherent nonlinear response in NVG observation enabled derivation of a mathematical model that provides the foundation for inverse gamma correction compensation. A prototype NIR-LED display was engineered with 1.25 mm pixel pitch and 1280 × 1024 resolution at 60 Hz refresh rate, achieving >90% uniformity and >2000:1 contrast. Subjective evaluations confirmed exceptional simulation fidelity. This system enables high-contrast, low-power NVG simulation for both full-flight simulators and urban low-altitude reconnaissance training systems, providing the first quantified analysis of NVG-LED nonlinear interactions and establishing the technical foundation for next-generation LED-based all-weather visual displays.

## 1. Introduction

Night vision goggles (NVGs) constitute a category of optoelectronic devices that amplify faint light signals through photon amplification and photoelectric conversion to produce visible imagery [1,2,3]. This capacity for generating clear images under darkness or low-light conditions enhances human night vision, enabling extensive deployment in military operations, security surveillance, law enforcement, and wilderness exploration. Through technological advancements, modern military systems widely employ third-generation (Gen III) NVGs, which feature Gallium Arsenide (GaAs) photocathodes. These photocathodes provide significantly higher sensitivity—particularly in the near-infrared (NIR) spectrum—compared to earlier generations (e.g., Gen II devices with multialkali photocathodes). This enhanced sensitivity translates into superior image resolution and markedly improved performance under low-light conditions, including starlight [4,5,6,7]. Particularly in aerial aviation, NVGs serve as critical equipment that augment pilots’ situational awareness and operational effectiveness during nighttime missions. By providing visual assistance at dusk or night, these significantly improve aircrew all-weather combat capabilities [8,9,10].

However, NVG deployment presents significant challenges for pilots. First, the constrained field-of-view (FOV)—typically ≈40°—limits pilots’ visual scanning capabilities during operations [11,12,13]. Second, monochromatic imaging output (typically green or white, determined by photocathode materials) combined with optical artifacts—halation, blooming, and shading—degrades image clarity [14,15]. This manifests as reduced image gradation, low contrast ratios, and increased visual interference. Such chromatic distortion and image degradation can trigger NVG-induced visual illusions, elevating the risk of spatial misjudgments that compromise flight safety. Finally, NVG usage increases pilot workload, accelerating fatigue development—a critical aviation safety concern [16,17,18,19,20]. Consequently, global air forces have implemented comprehensive NVG training programs to address these challenges, focusing on developing pilot proficiency in NVG operation, adapting to NVG-induced visual perception changes, and mitigating physiological impacts.

Following the initial NVG deployment in the 1970s, the United States has established dedicated NVG training research that evolved from early studies on ground troop effectiveness to sophisticated, aviation-centric curricula. These curricula encompass soldier competency in NVG operation for nocturnal missions, equipment integration methods, and distance estimation enhancement under NVG conditions [21,22]. Simultaneously, NATO Standardization Agreement STANAG 7147 governs NVG training standards through a structured framework: Initial Qualification Training (ground), Recurrent Training (ground), and Flight Training. The Initial Qualification phase integrates theoretical instruction with hand-on NVG configuration/inspection and simulator-based training [23]. Globally, the military aviation community recognizes ground-based simulated NVG training—particularly live goggle drill—as critically valuable. This training enhances mission readiness, operational familiarity, and flight perception adaptation, substantially improving aviation safety [16,24,25,26,27,28,29,30,31,32].

Consequently, devices capable of delivering realistic video imagery for NVGs have become critical for effective ground-based training. Currently, ground-based NVG simulation training primarily relies on two methodologies, (1) Image Generator (IG) direct simulation, which renders synthetic NVG-like imagery—characterized by monochrome green/white, noise and halos—on conventional displays [33], and (2) physical simulation, in which trainees wear actual NVGs and view a specialized display system engineered to stimulate the image intensifier tube of the goggles [34]. While IG simulation offers advantages in implementation simplicity and minimal hardware requirements, it fundamentally fails to replicate the critical physical interactions and perceptual characteristics inherent in real-world NVG operations. Specifically, it does not accurately reproduce the FOV, spatial awareness constraints imposed by helmet-mounted devices, authentic optical artifacts (e.g., dynamic halation), or the realistic visual experience provided through optical eyepieces. Consequently, IG simulation falls short in preparing pilots for the operational challenges of NVG handling and the visual–spatial adaptations required during actual missions. In contrast, physical simulation using real NVGs delivers the most immersive and operationally relevant training experience. It faithfully replicates the complete sensory and physical state of a pilot observing the external environment through NVGs during flight. This includes accurate simulation of the device’s spectral response, nonlinear luminance conversion, optical distortions, and system-specific artifacts, as well as realistic viewing posture/environmental constraints. Consequently, physical simulation enables comprehensive training in physical interaction, perceptual fidelity, and physiological adaptation. However, the adoption of physical simulation introduces a significant technical challenge: the high-fidelity design and implementation of the display system serving as the NVG stimulator source. Crucially, developing a single display system capable of supporting all-weather flight simulation—including daytime and nighttime scenarios—for a unified platform remains a significant technical challenge. Current research indicates that projection-based display systems––primary solution for NVG compatible flight simulators––integrate auxiliary light sources to simultaneously present visible imagery and stimulate NVG image intensifiers (e.g., Barco FS40/FS70/FS400 series) [34,35,36,37]. Despite advantages in retrofit flexibility, continuous operability, and display integration, such projection systems fundamentally grapple with contrast ratio limitations. Night scene simulation often suffers from diminished image clarity and loss of grayscale resolution due to intrinsic technological constraints [38,39].

Notably, LED-based visual display systems have been documented in flight simulation training applications [40]. While they deliver exceptional visual performance through superior luminance and contrast, their implementations have been confined to daytime scenario simulation; no NVG-compatible configurations have been documented. To address the limitations of projection technology––particularly its insufficient contrast for authentic NVG simulation––and to expand the applicability of LED-based displays into the critical domain of night vision training, this paper proposes a novel direct-view LED display system configured as an NVG imaging stimulator source. The core motivation behind developing this NIR-LED direct-view system is to harness the inherent advantages of LED technology—such as high contrast ratios, fast response times, and precise luminance control—to deliver a significantly higher-fidelity image stimulation source for NVGs. This directly addresses a critical gap in existing solutions for authentic NVG simulation training. Simultaneously, this research aims to establish the theoretical foundation and technical pathway for extending advanced LED-based dome visual display systems—currently restricted to daytime simulation—into the realm of all-weather training applications. Leveraging inherently high contrast ratios and rapid response characteristics, this system enables accurate night scene reproduction, particularly during dynamic scenario transitions, achieving unprecedented simulation fidelity. Such advancements directly contribute to enhanced pilot training efficacy during ground-based NVG exercises. Through the development of this system, we investigate the response characteristics of NVGs to NIR-LEDs, validating the feasibility and identifying key challenges of using LED display technology as an NVG stimulation source under ultra-low-light conditions. We characterize and model the nonlinear response of NVGs and propose an inverse gamma correction method to accurately compensate for its impact on display performance—significantly improving simulation fidelity, as confirmed by subjective evaluations of NVG imagery. Furthermore, this work presents the first nonlinear interaction mechanism between NVGs and LEDs, establishing a critical technical foundation for the development of next-generation, all-weather LED-based visual display systems.

This paper is structured as follows: Section 2 presents the design and implementation of the NIR-LED direct-view display, with particular emphasis on the spectral selection process that establishes 940 nm as the optimal wavelength. Section 3 characterizes the inherent nonlinear response of NVG image intensifiers and proposes an inverse gamma correction method for compensation. Section 4 quantifies display performance through uniformity and contrast measurements, complemented by visual validation using authentic night vision scenes, collectively validating its operational viability.

## 2. Design of an NIR-LED Direct-View Display

### 2.1. LED Spectral Selection for Night Vision Simulation

Spectrum selection proves critical for engineering direct-view LED displays in NVG-compatible training applications, driven by dual imperatives:

(1) The display must simulate human nocturnal vision to create authentically low-illuminance conditions, enabling non-NVG-equipped pilots to experience authentic nocturnal immersion.
(2) LED emission must both trigger NVG detection and accurately replicate NVG spectral responses to natural night-sky irradiance.

Consequently, optimal LED spectral selection necessitates integrated consideration of the interrelationship between human visual perception, NVG spectral response characteristics, and night-sky radiation profiles. To establish the analytic framework, we referenced three datasets: the photopic luminosity function standardized by the Commission Internationale de l’Éclairage (CIE) for human spectral sensitivity [41], the NVG response characteristics codified in U.S. Department of Defense specification MIL-STD-3009 [42], and the night-sky irradiance distributions documented by Vatsia et al. [43]. Comparative spectral diagrams (380–1100 nm wavelength, Figure 1) delineate correlations between night-sky radiation, human perceptual thresholds, and Generation III NVG spectral sensitivity.

**Figure 1:**
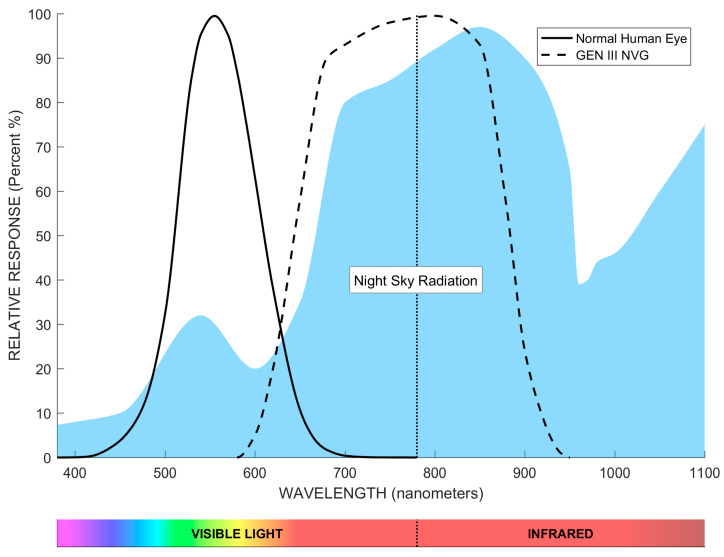
Spectral response profiles of human vision and Generation III NVGs.

As evidenced by the blue-shaded night-sky radiation profile in Figure 1, natural nocturnal radiation intensity predominantly concentrates in the near-infrared (NIR) spectrum beyond 700 nm. Consequently, NVGs are engineered to achieve peak responsivity within the NIR band. For instance, third-generation devices exhibit peak spectral response between 580 nm and 950 nm. Therefore, NIR-band LEDs were chosen as the optimal spectral solution to simultaneously achieve the following objectives:

(1) Match the natural night-sky irradiance spectrum (dominant above 700 nm) [35,36];
(2) Align with NVG peak responsibility (580–950 nm) [1,4,5,42];
(3) Avoid visual interference for unaided human observers during nighttime training scenarios (human vision has negligible sensitivity beyond 700 nm).

This triple-alignment strategy—spanning night-sky physics, NVG sensitivity, and human physiological constraints—ensures ecological valid nocturnal simulation and operational compatibility with dual-user (NVG-equipped/unaided) training.

**Figure 2:**
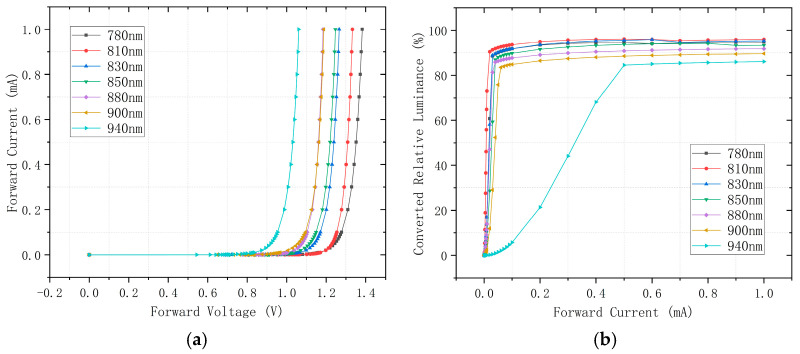
Current-voltage characteristics and irradiance-to-current response curves of ultra-low current NIR-LEDs: (**a**) current-voltage characteristics; (**b**) irradiance-to-current response under NVG-coupled measurement.

To identify optimal wavelengths, we characterized seven LEDs (780–940 nm, manufactured by Shenzhen Baoyoute Industrial Development Co., Ltd., Shenzhen, China) with identical package dimensions, total radiant power, and viewing angles using an ADCMT 6146 precision current source (ADCMT Corporation, Saitama, Japan; voltage accuracy: ±1 μV, current accuracy: ±0.1 μA) to apply forward voltage and a Gwinstek GDM-8261 digital multimeter (6½-digit) for current measurement (accuracy: ±0.1 μA) by evaluating their current-voltage behavior under ultra-low current conditions (μA-range) and measuring the corresponding NVG-observed luminance–current relationships under photon amplification. Results in Figure 2a reveal consistent characteristics across wavelengths; current exhibits exceptional growth beyond a forward voltage threshold, confirming significant challenges in achieving high-gray-level display at low luminance values. When observed through third-generation NVGs (Onick NVG-D3, Onick Optics (Wuhan) Co., Ltd.,Wuhan, China) (with a 42° field of view, a photocathode radiant sensitivity of 500–600 μA/lm, 1× magnification, a limiting resolution of 51–57 lp/mm, and a GaAs photocathode material), the photon-amplified luminance exhibited heightened sensitivity to current variations. Figure 2b demonstrates luminance saturation at currents as low as 20 μA (810 nm) and 60 μA (900 nm), indicating extreme difficulty in rendering high-bit-depth grayscale imagery. Critically, these saturation currents inversely correlate with NVG spectral sensitivity shown in Figure 1: wavelengths aligning with sensitivity peaks saturate at lower currents. The 940 nm LED demonstrates saturation at 500 μA, where its attenuated spectral responsivity enables precise current gradation control. This facilitates superior low-brightness high-gray-level rendering, making it particularly advantageous for generating high-fidelity grayscale imagery in NVG-compatible direct-view displays.

Notably, while human vision perceives 380–780 nm wavelengths, experimental characterization revealed that near-infrared LEDs emit visible red light (“red glow” effect) when driven beyond threshold currents. This phenomenon poses critical operational risks in direct-view displays integrating millions of NIR-LEDs, potentially inducing pilot physiological discomfort and psychological distress—manifested as tension, irritability, and impulsiveness. To mitigate this risk, we quantified red glow thresholds for all seven NIR-LEDs within a darkroom environment (<10^−5^ lx illuminance). Table 1 details the minimum currents required to elicit perceptible red glow, demonstrating progressive threshold current elevation with wavelength; 780 nm LEDs exhibited visible glow at 35 μA, 810–850 nm variants required μA-range currents, 880/900 nm devices reached thresholds at 2 mA, while 940 nm LEDs showed negligible emission. This current threshold was not recorded in Table 1 for 940 nm LEDs because, during our experiments, we applied currents up to the maximum allowable limit of 25 mA without observing the “red glow” phenomenon. Further increases in current would have risked permanent damage to the devices. Therefore, under normal operating conditions, the red glow was not observed for 940 nm LEDs, and no corresponding threshold value is listed in Table 1. Consequently, 940 nm LEDs emerge as the optimal solution, delivering dual advantages, enabling low-brightness high-gray-level rendering while eliminating red glow interference in NVG simulation displays.

### 2.2. System Hardware Design

**Figure 3:**
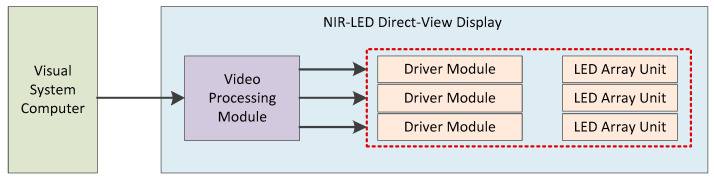
System hardware architecture block diagram.

Building upon the established spectral and LED selection rationale, this section details the implementation of the system hardware design. For direct-view displays utilizing 940 nm NIR-LEDs (Dongguan Lanjin Optoelectronics Co., Ltd., Dongguan, China), this work proposed an optimized hardware architecture comprising three core components: a video processing module executing RGB-to-grayscale conversion, luminance regulation, gamma correction, image segmentation/distribution, and video interface adaptation; a driver module paring image data into scanning control signals; and an LED array unit (Figure 3) comprising matrix scan driver ICs and LED devices that convert control signals into PWM signals. In practical implementations, display panels are composed of multiple tiled LED array units. A single driver module typically controls multiple arrays, with its load capacity determined by pixel count, grayscale depth, and refresh rate. Video processing module configuration scales with display resolution, requiring only one module for systems at or below 4K resolution (3840 × 2160).

**Figure 4:**
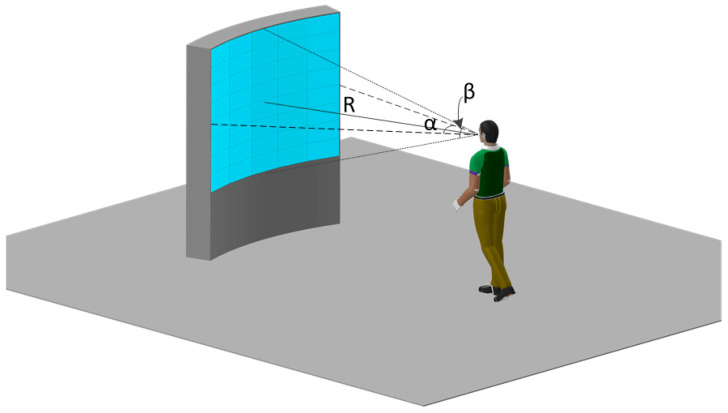
Rendering of the LED direct-view display prototype.

During simulation training with pilots positioned 2.5–3.5 m from the display, this design ensures high-definition imagery by first determining pixel pitch based on spatial resolution (arcmin/pixel), then defining resolution, and finally establishing screen dimensions. Although 1 arcmin/pixel (60 ppd) delivers optimal visual acuity, established LED display engineering practice confirms resolutions ≤2 arcmin/pixel (30 ppd) maintain excellent visual quality while substantially reducing hardware resource demands and power consumption [44,45,46]. Accordingly, this implementation adopted a 1.25 mm pixel pitch with 1280 × 1024 resolution. For a cylindrical screen configuration of radius R = 2500 mm, this yields a horizontal field of view (HFOV) of 32.62° and vertical field of view (VFOV) of 28.72°, achieving a calculated spatial resolution of 1.72 arcmin/pixel––delivering high-definition NVG-compatible imagery. The finalized NIR-LED direct-view display rendering is demonstrated in Figure 4.

## 3. NVG Response Characteristics and Gamma Correction for NIR-LED Direct-View Displays

### 3.1. Modeling of NVG Response Characteristics to NIR-LED Displays

**Figure 5:**
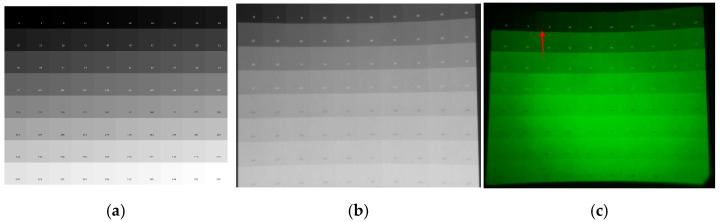
Grayscale test patterns of the NIR-LED direct-view display: (**a**) original grayscale test chart; (**b**) infrared camera captured image; (**c**) NVG-captured image.

Functioning as a monochromatic system, the developed NIR-LED direct-view display prioritized grayscale reproduction fidelity—particularly under NVG observation—as its primary performance metric. Accordingly, display performance was evaluated using a 0–255 grayscale step wedge test pattern comprising 80 discrete blocks. The gray level of these blocks increased incrementally by steps of three or four from 0 to 255. As illustrated in Figure 5b, IR-captured images demonstrate close conformity to original images, validating high-fidelity grayscale resolution. The IR images were captured using a commercially available infrared camera (Model IUA8300KME, Tupu Photoelectric Technology Co., Ltd., Hangzhou, China). This camera is equipped with a CMOS image sensor having a spectral response range of 400 nm–1000 nm, a pixel size of 2.0 µm × 2.0 µm, and an optical format of 1/1.8”. It offers a 12-bit dynamic range and captures images at a resolution of 3840 × 2160 pixels, with a frame rate of 45 frames per second (fps). However, as shown in Figure 5c, under NVG observation, the on-screen grayscale transition no longer progresses smoothly as in Figure 5a. Instead, beginning at gray level 6, the brightness of the blocks increases sharply (indicated by the red arrow). By the time the gray level reaches 129—corresponding to the initial block of the fifth row—the luminance effectively saturates, making adjacent blocks visually indistinguishable. Consequently, establishing an NVG response model is essential for developing effective luminance compensation solutions.

**Figure 6:**
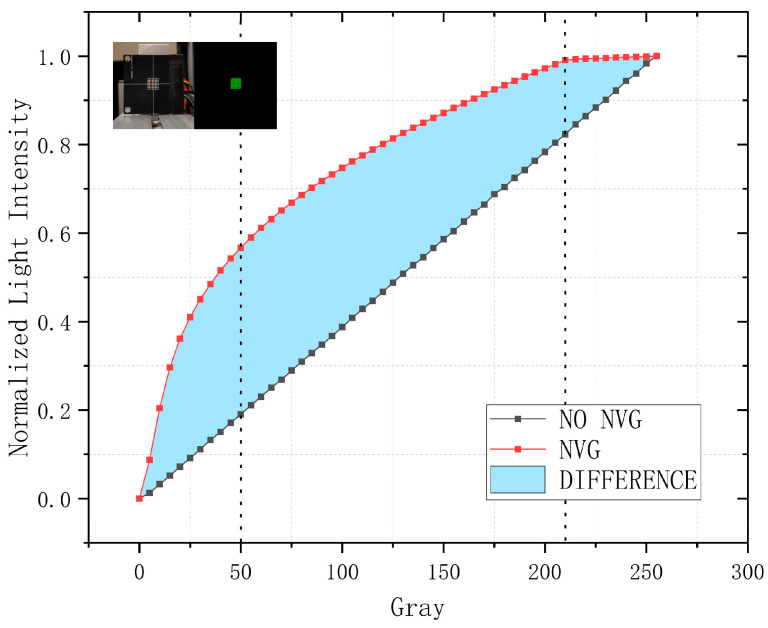
Nonlinear response curve of NVGs.

LED display operation adheres to pulse-width modulation (PWM) principles governed by $L_{\mathrm{out}}=L_{max}\frac{\tau }{T}$, where $L_{\mathrm{out}}$ denotes LED luminance, $L_{max}$ represents full-duty-cycle luminance, T is PWM period, and τ designates on-time duration. With constant $L_{max}$ and T, luminance $L_{\mathrm{out}}$ varies linearly with τ, establishing proportionality to displayed grayscale levels. To characterize grayscale response under NVG observation with identical PWM driving conditions, we conducted an experiment where an FPGA controller drove an 8 × 8 LED matrix (1.25 mm pitch, 940 nm) with fixed T = 51,200 ns while increasing τ from 0 to 51,200 ns in 200 ns steps (generating 256 grayscale levels; measurements at 51 points). Infrared irradiance was measured in a darkroom using an Instrument Systems GmbH (München, Germany) CAS140CT-156 spectroradiometer with EOP146 probe (spectral response range: 300–1100 nm), while NVG output luminance was quantified via Konica Minolta CA-310 color analyzer (spectral response range: 380–780 nm). Finally, PWM-controlled luminance values were mapped to grayscale levels with normalized irradiance and luminance data, producing the results shown in Figure 6.

Both curves in Figure 6 exhibit monotonic increase from 0 to 1, yet the NVG-observed curve consistently exceeds direct measurements in normalized values, confirming NVGs’ irradiance amplification throughout the input range. This enhancement is particularly pronounced at low irradiance (grayscale 0–50), indicating enhanced NVG responsivity under diminished irradiance. Conversely, in high-irradiance regions (grayscale 210–255), NVG-observed outputs demonstrate minimal increments with progressive stabilization, confirming asymptotic convergence toward saturation in this region. Mid-irradiance zones approximate linearity. These observations establish that NVGs exhibit inherent non-linear response characterized by low-irradiance enhancement and high-irradiance compression.

For effective compensation of NVG-induced effects, we developed mathematical models to precisely characterize their response. Based on the NVG response curve characteristics in Figure 6, we proposed a preliminary power-law mapping model expressed as:

(1) $$y=cx^{\gamma }$$

where x and y denote input and output grayscale values, respectively, and c and γ are positive constants. For simplify fitting, we assumed c=1 and employed nonlinear least-squares regression to fit $y=x^{\gamma }$. Given data points $\left(x_{i},y_{i}\right)$ (excluding (0,0)), the objective minimizes residual sum of squares by optimizing γ. The objective function is defined as:

(2) $$\underset{\gamma }{min}S(\gamma )=\sum_{i=1}^{n}{\left(y_{i}-x_{i}^{\gamma }\right)}^{2}$$

where n denotes the number of data points. To establish mathematical optimality, we verified the first-order condition by differentiating the objective function S(γ):

(3) $$\frac{\partial S}{\partial \gamma }=-2\sum_{i=1}^{n}\left[(y_{i}-x_{i}^{\gamma })\cdot x_{i}^{\gamma }lnx_{i}\right]$$

The optimal solution $\gamma^{*}$ satisfies the vanishing gradient condition. We therefore implemented the Gauss–Newton method to solve this nonlinear least-squares problem [47,48], with parameter update rule:

(4) $$\gamma^{\left(k+1\right)}=\gamma^{\left(k\right)}-\frac{J^{Τ}r}{J^{Τ}J}$$

where k denotes iteration index, $r={\left[r_{1},\cdots ,r_{n}\right]}^{Τ}$ represents the residual vector with components $r_{i}=y_{i}-x_{i}^{\gamma^{\left(k\right)}}$, and $J={\left[J_{1},\cdots ,J_{n}\right]}^{Τ}$ the Jacobian vector defined by $J_{i}=-x_{i}^{\gamma^{\left(k\right)}}lnx_{i}$. Numerical solution in MATLAB R2016a used initial estimate $\gamma^{\left(0\right)}=0.5$, maximum 100 iterations, and convergence tolerance $\left|\gamma^{\left(k+1\right)}-\gamma^{\left(k\right)}\right|<10^{-8}$ for successive parameter increments. The fitted solution yielded $\gamma_{o}\approx 0.35$ with residual sum of squares S(γ)=0.1047, establishing the final model as $y=x^{0.35}$.

### 3.2. Gamma Correction for Direct-View Displays Compensating NVG Nonlinear Response Characteristics

**Figure 7:**
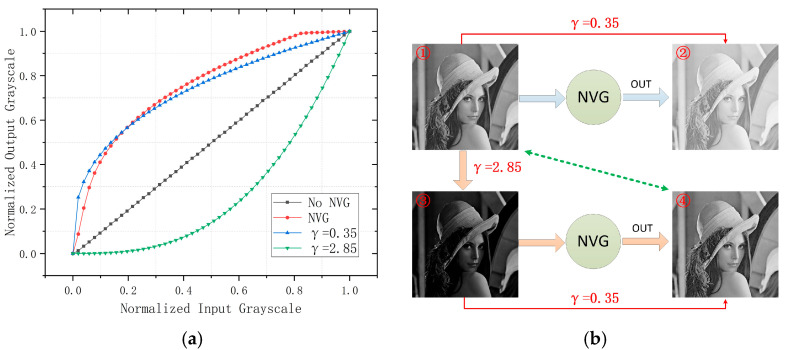
Gamma compensation schematic: (**a**) nonlinear response curve of NVGs versus gamma compensation profiles; (**b**) block diagram of inverse gamma correction implementation. (① Original test chart; ② rendering after γ = 0.35 compensation; ③ rendering after γ = 2.85 compensation; ④ output after applying γ = 0.35 to 3).

Figure 7a demonstrates close alignment between the fitted curve and empirical NVG response. In digital image processing, the exponent in Model (1) is conventionally designated as gamma [49,50]. The NVG response effectively applies gamma correction with γ=0.35 to input images, as evidenced by the Figure 7b (①→②) transformation. This gamma adjustment induces significant output luminance elevation. To counteract this effect, we implemented inverse gamma transformation by setting the exponent to $\frac{1}{\gamma }$ in Model (1). For the NVG response discussed herein, calculated gamma compensation with γ=2.85 generates the Figure 7b (①→③) transformation. Subsequent NVG observation of image ③ produces output ④, achieving perceptual equivalence with the original image ① (as indicated by the green arrow in the figure). Due to equipment availability constraints, this study focused exclusively on the NVG-D3 night vision goggle. As a result, the proposed inverse gamma correction method remains relatively straightforward. Future research should extend validation to a broader range of night vision devices to gather more comprehensive data. By integrating these findings with dynamic gamma correction models and JND quantification methods—such as those proposed by Qian et al. [51]—it may be possible to develop a more universal and accurate mathematical compensation framework. Such a model would provide a stronger theoretical foundation for generating realistic night vision imagery using NIR-LED direct-view displays.

**Figure 8:**
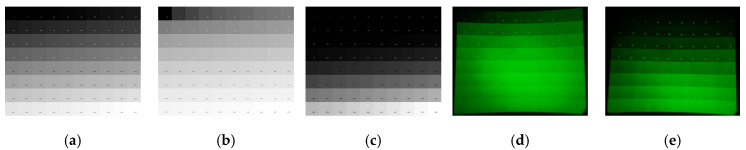
Gamma compensation comparative results: (**a**) original grayscale test chart; (**b**) γ = 0.35 compensated rendering; (**c**) γ = 2.85 compensated rendering; (**d**) NVG-captured image of original display; (**e**) NVG-captured image after γ = 2.85 screen compensation.

We further validated grayscale rendering fidelity on the NIR-LED direct-view display by implementing gamma compensation at γ = 2.85. Results in Figure 8 demonstrate significant similarity between images (b) and (d), both exhibiting compressed shadow regions and expanded highlight zones. These characteristics align with the effects of γ ≈ 0.35 transformation observed during NVG processing. Crucially, the output (e) replicates the perceptual gradation of reference (a), while clearly resolving each row of grayscale blocks—unlike the saturation and loss of contrast observed beyond the fifth row in (d). Simultaneously, it preserves low-luminance grayscale differentiation to enhance dark-scene detail visibility. These findings verify that γ = 2.85 compensation prior to NVG observation achieves perceptual equivalence to the original scene, thereby demonstrating effective mitigation of NVG nonlinearity through inverse gamma transformation.

## 4. Experimental Results

### 4.1. Display Uniformity and Contrast Ratio Characterization

**Figure 9:**
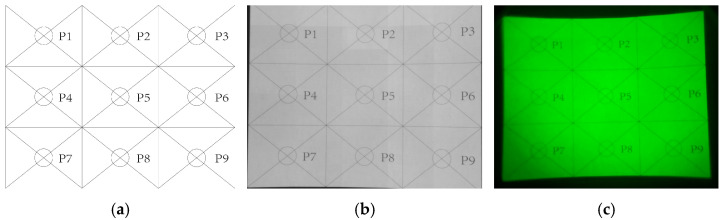
Display uniformity test patterns: (**a**) uniformity test pattern; (**b**) infrared camera-captured display image; (**c**) NVG-captured display image.

To quantitatively evaluate the proposed NIR-LED direct-view display performance, we assessed two critical parameters: display uniformity and contrast ratio. Uniformity testing followed IEC 62922 specification [52] under full-field illumination at maximum brightness, with luminance measured at nine positions in a standardized 3 × 3 grid. Luminance uniformity was calculated as:

(5) $$U=\left[1-\frac{L_{max}-L_{min}}{L_{max}+L_{min}}\right]\times 100\%=\left[2\times \frac{L_{min}}{L_{max}+L_{min}}\right]\times 100\%$$

where U denotes display uniformity and $L_{max}$ and $L_{min}$ represent maximum and minimum luminance among the nine test points, respectively. Given the display’s 940 nm emission wavelength, infrared irradiance measurement replaced luminance for performance characterization. Darkroom measurements employed a CAS140CT-156 spectroradiometer with EOP146 probe in direct screen contact, with test images depicted in Figure 9. Figure 9a shows the designed test pattern (1280 × 1024), matching the resolution of the NIR-LED direct-view display to ensure pixel-to-pixel rendering. Figure 9b presents the infrared camera capture, confirming high sharpness and faithful imaging under point-to-point mapping. Despite lower resolution due to sensor limitations, Figure 9c shows that the NVG-resolved image remains discernible, with clear edges and legible text, indicating adequate resolution performance for practical NVG simulation.

Testing data from points P1 to P9 identified P2 and P8 as the maximum and minimum irradiance points, respectively. Substituting these values into Equation (5) (Table 2) yields a display uniformity of 92.12% for the NIR-LED direct-view screen. This high uniformity confirms excellent spatial consistency, generally eliminating luminance compensation requirements in practical applications.

**Figure 10:**
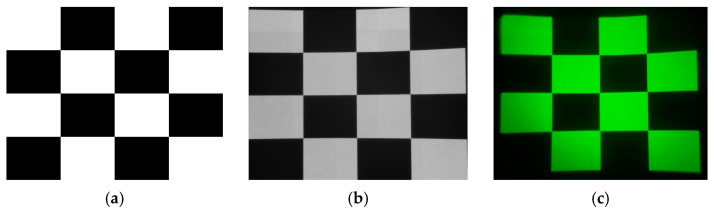
Display contrast test patterns: (**a**) contrast test pattern; (**b**) infrared-camera-captured display image; (**c**) NVG-captured display image.

Higher display contrast ratios expand dynamic range representation—particularly crucial for low-light night vision scenarios where enhanced contrast preserves shadow detail while preventing image washout. Following IEC 61947-1 Electronic projection—Measurement of performance standards [53], we evaluated the NIR-LED direct-view screen using a 16-cell checkerboard test pattern comprising eight white and eight black alternating squares (Figure 10). The contrast ratio is calculated as:

(6) $$CR=\frac{Avg(L_{white})}{Avg(L_{black})}$$

where $\;Avg(L_{white})$ and $Avg(L_{black})$ denote the average irradiance of all white and black squares, respectively. Infrared irradiance measurements yielded $Avg\left(L_{white}\right)=9.138\;mW/m^{2}\cdot nm$ and $Avg\left(L_{black}\right)=4.195\;uW/m^{2}\cdot nm$, producing a screen contrast ratio of CR≈2178:1.

### 4.2. Night Vision Simulation Effectiveness

**Figure 11:**
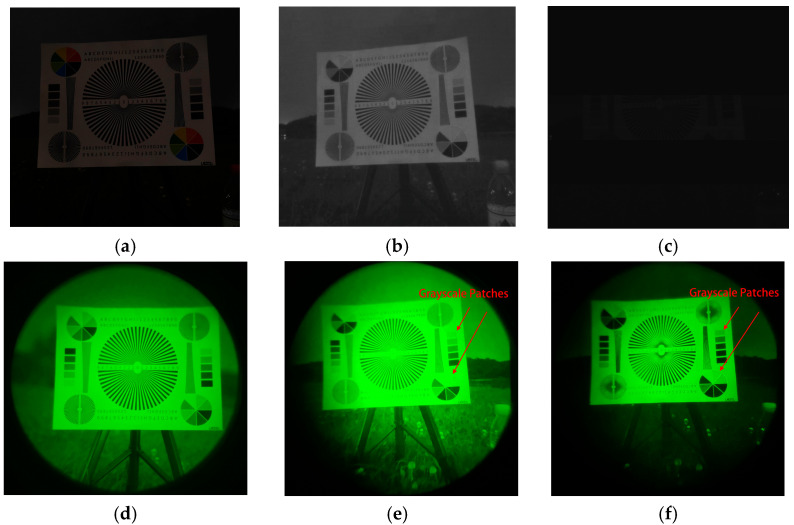
Night vision scene rendering comparison of NIR-LED display: (**a**) reference night scene 1; (**b**) infrared-camera-captured display output (γ = 1); (**c**) infrared-camera-captured display output (γ = 2.85); (**d**) NVG-captured reference scene 1; (**e**) NVG-captured display output (γ = 1); (**f**) NVG-captured display output (γ = 2.85).

To further validate the NIR-LED display’s night vision rendering performance, we conducted experiments using an image quality test chart and an urban night scene. The reference image (Figure 11a), captured by a visible-light camera under night-sky conditions, features line patterns for spatial resolution assessment, text elements for evaluating legibility, and grayscale patches for tonal reproduction evaluation. Simultaneously, NVG-captured imagery of the same test chart under identical conditions (Figure 11d) demonstrates NVGs’ capability to resolve fine details and grayscale gradations. For display validation, Figure 11a content was rendered on-screen at gamma settings γ=1 and γ=2.85  and observed through NVGs. Key observations were as follows:

- γ=1 rendering (Figure 11e) exhibits highlight clipping in bright regions (as arrow-indicated), compromising grayscale discrimination.
- γ=2.85 rendering (Figure 11f) maintains discernible details at reduced luminance, while preserving grayscale separation.

Infrared camera captures confirmed that γ=2.85 implementation achieved targeted luminance attenuation. These results corroborate Section 3.2 conclusions on NVG-compatible gamma compensation efficacy.

**Figure 12:**
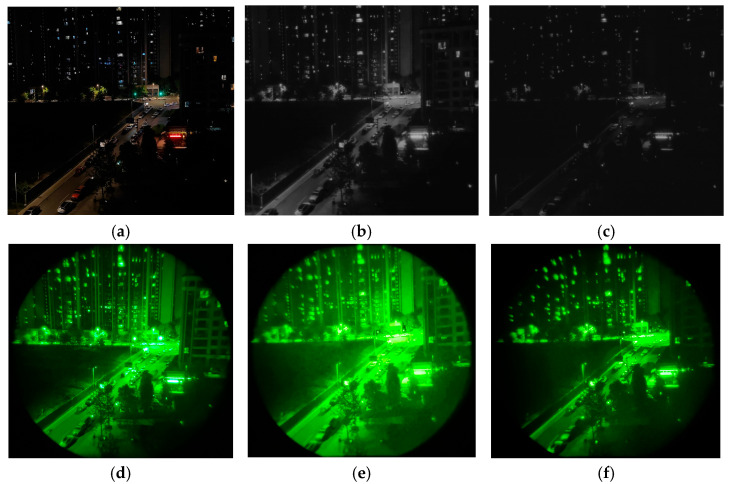
Night vision scene rendering comparison of NIR-LED display: (**a**) reference night scene 2; (**b**) infrared-camera-captured display output (γ = 1); (**c**) infrared-camera-captured display output (γ = 2.85); (**d**) NVG-captured reference scene 2; (**e**) NVG-captured display output (γ = 1); (**f**) NVG-captured display output (γ = 2.85).

We validated the protocol using an urban night scene (Figure 12). Direct NVG observation of the actual scene (Figure 12d) achieved excellent clarity and contrast, resolving fine details in low-illuminance areas. When displaying the scene on the NIR-LED screen under NVG observation:

- γ=1 rendering (Figure 12e) exhibits luminance elevation in shadow regions (foliage, lawns, and building facades/windows) combined with expanded highlight saturation zones, collectively inducing resolution degradation.
- γ=2.85 rendering (Figure 12f) demonstrates contrast characteristics approaching the reference output (Figure 12d), maintaining highlight detail clarity (vehicles and streetlights) despite slightly reduced overall sharpness.

Thus, γ=2.85 output achieves superior scene fidelity relative to actual night vision observation.

## 5. Conclusions

This study pioneers the application of LED-based direct-view displays for pilot night vision goggle (NVG) simulation training. Through comprehensive investigations of LED spectral optimization, NVG response characteristics to NIR-LED displays, and compensation methodologies for NVG nonlinearity, we developed a 940 nm NIR-LED display prototype featuring 1.25 mm pixel pitch, 1280 × 1024 resolution, 60 Hz refresh rate, 92.12% uniformity, and 2178:1 contrast ratio. Compared to traditional NVG simulation displays, the developed NIR-LED direct-view system achieves a significantly higher contrast ratio of 2178:1, greatly enhancing the realism of simulated night vision scenes. Furthermore, this work presents the first successful implementation of NIR-LEDs as an image excitation source for NVG simulation, offering a novel approach for night vision simulator display systems. The proposed inverse gamma transformation effectively compensates for NVG nonlinear response, with efficacy validated through qualitative analysis. Comparative assessments demonstrate that NVG observation of this NIR-LED display produces visual perceptions closely approximating direct observation of actual night scenes, confirming its applicability for pilot NVG simulation training. Furthermore, this work establishes a technical foundation for integrated visible-light/NIR-LED displays capable of supporting all-weather simulation training systems under both daylight and nocturnal conditions. Collectively, this system resolves critical fidelity challenges in NVG simulation imagery for pilots, thereby enhancing flight safety during NVG-assisted night operations, and provides a basis for developing next-generation LED-based all-weather display systems.

## Figures and Tables

**Table 1:** Threshold currents for “red glow” effect in NIR-LEDs.

Wavelength (nm)	780	810	830	850	880	900	940
Threshold currents (μA)	35	75	300	400	2000	2000	--

Notes: (1) These data may exhibit variations due to differences in observer visual perception. (2) “--” indicates not visible or undetected.

**Table 2:** Infrared irradiance test data of NIR-LEDs.

Test Point	P1	P2	P3	P4	P5	P6	P7	P8	P9
Infrared irradiance ($mW/m^{2}\cdot nm$)	9.285	9.741	9.265	8.502	8.843	9.276	8.833	8.318	9.372

## Data Availability

The datasets generated during this study are available from the corresponding author on reasonable request.
